# Comparative Meta-Analysis of the Efficacy of Once-Daily Fluticasone Furoate 100 µG Versus Twice-Daily Fluticasone Propionate 250 µG in Adolescents and Adults with Persistent Asthma

**DOI:** 10.1007/s00408-017-0041-2

**Published:** 2017-07-28

**Authors:** Ryan Tomlinson, Daniel Parks, Alan Martin

**Affiliations:** 1 Respiratory Clinical Discovery, GSK, 709 Swedeland Road, King of Prussia, Upper Merion, PA 19406 USA; 2 Value Evidence Analytics, GSK Upper Providence, 1250 South Collegeville Road, Collegeville, PA 19426 USA; 3 Value Evidence Analytics, GSK, 1-3 Iron Bridge Rd, Uxbridge, UB11 1BT UK

**Keywords:** Asthma, Forced expiratory volume in 1 s, Fluticasone furoate, Fluticasone propionate, Inhaled corticosteroid, Meta-analysis

## Abstract

Fluticasone furoate and fluticasone propionate are recommended options for prophylactic maintenance treatment of persistent asthma. Using data from two previous clinical studies (GSK studies: FFA109685/NCT00603278, FFA112059/NCT01159912), this meta-analysis compared change from baseline in clinic visit mean trough forced expiratory volume in 1 s (FEV_1_) with fluticasone furoate 100 µg once-daily (FF100) versus fluticasone propionate 250 µg twice-daily (FP250) in adolescents and adults with persistent asthma. Using a DerSimonian–Laird random-effects model (primary meta-analysis), there was no statistically significant difference between FF100 and FP250 in change from baseline in trough FEV_1_ (−1.7 mL [95% CI −80.4, +77.0], *p* = 0.9664) and FF100 was non-inferior to FP250. Supporting analyses using least squares mean and fixed-effects model approaches produced similar findings. In this analysis, FF100 and FP250 demonstrated a comparable treatment effect on trough FEV_1_ in patients aged ≥12 years with persistent asthma; however, results interpretation should consider study design and methodological limitations.

## Introduction

The inhaled corticosteroids (ICS) fluticasone furoate (FF) and fluticasone propionate (FP) are among the recommended options for the prophylactic maintenance treatment of persistent asthma [1]. While both belong to the glucocorticoid class, FF and FP are structurally distinct drugs with distinct physiochemical properties [2]. The structure of FF confers higher affinity for both nasal and lung tissue compared with FP, affording improved lung residency and once-daily efficacy in patients with asthma [2].

With its indicated once-daily dosing [3], FF may offer advantages over twice-daily dosing with FP [4] in terms of patient convenience and treatment adherence. The efficacy and safety of FF 100 µg once-daily (FF100) has been demonstrated in two randomised, placebo-controlled trials in patients aged ≥12 years with persistent asthma uncontrolled by low-/mid-dose ICS [5, 6]. In these studies, FF100 and FP 250 µg twice-daily (FP250) both demonstrated significant improvements over placebo in pre-specified lung function endpoints. Furthermore, FF100 exhibited similar lung function effects to FP250; however, neither study was powered to directly compare the two treatments [5, 6]. This meta-analysis compared change from baseline in clinic visit mean trough forced expiratory volume in 1 s (FEV_1_) with FF100 and FP250 in patients aged ≥12 years with persistent asthma.

## Methods

This analysis (GSK study 204521) was conducted at the request of a health technology assessment body as part of an FF appraisal process. As the purpose was not to include indirect evidence in a network meta-analysis, no systematic review was necessary.

The meta-analysis combined data derived from the FF100 and FP250 arms of two independent, randomised, placebo-controlled, parallel-group clinical studies in patients aged ≥12 years with persistent asthma, who prior to the study were receiving a stable dose of ICS (GSK studies FFA109685; NCT00603278 [5] and FFA112059; NCT01159912 [6]). In both studies, FF100 was compared to placebo and FP250 was included only as a reference active-control arm; no prior statistical comparisons were made between FF100 and FP250.

The primary objective of this meta-analysis was to compare FF100 and FP250 in mean change from baseline in pre-dose trough FEV_1_ at the primary endpoint analysis time point (8 weeks in FFA109685; 24 weeks in FFA112059). In both studies, the primary efficacy analysis was conducted in the intent-to-treat (ITT) population using a last observation carried forward (LOCF) approach for imputation of missing data. Statistical analysis of the primary endpoint was comparable between the two trials, both using analysis of covariance (ANCOVA) with covariates of baseline, region, sex, age, and treatment. The meta-analysis was conducted using a frequentist approach, employing a DerSimonian–Laird random-effects model [7] to statistically combine the results for the mean difference in change from baseline in pre-dose trough FEV_1_ from the individual trials. Statistical heterogeneity was assessed using the Cochran *Q* Chi square test and the *I* ^*2*^ statistic [8]. Non-inferiority of FF100 to FP250 was evaluated using a non-inferiority margin of 200 mL, an accepted minimally important clinical difference in FEV_1_ in asthma [9–11]. Separate supporting analyses incorporating a least squares (LS) mean approach and using a fixed-effects model were also carried out. All analyses were conducted using the ‘meta’ package in R v3.1.1 (R Foundation for Statistical Computing, Vienna, Austria).

## Results and Discussion

Data from 433 patients included in the ITT populations of the FF100 and FP250 arms of the two clinical studies were combined for this meta-analysis (FFA109685: FF100 *n* = 105, FP250 *n* = 100; FFA112059: FF100 *n* = 114, FP250 *n* = 114). Baseline characteristics were comparable between treatment arms within and across the two studies, except for a higher rate of previous ICS + long-acting β_2_ agonist treatment in study FFA109685.

**Fig. 1:**
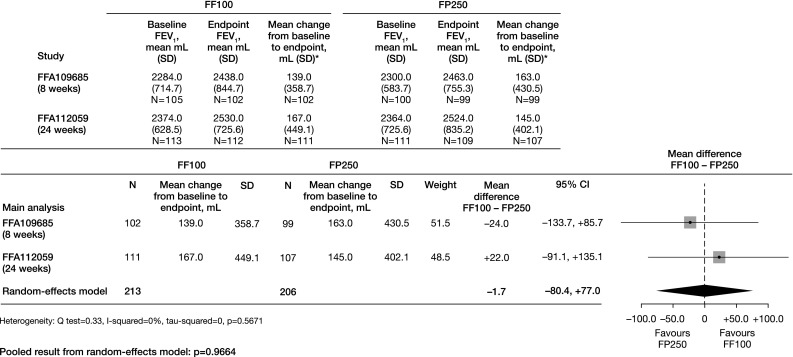
Comparative meta-analysis of FF100 and FP250 in mean change from baseline in trough FEV_1_ (random-effects model) *Patients with FEV_1_ values both at baseline and at the time point of the primary endpoint assessment (8 weeks in FFA109685; 24 weeks in FFA112059) *CI* confidence interval, *FEV*_*1*_ forced expiratory volume in 1 s, *FF100* fluticasone furoate 100 µg once-daily, *FP250* fluticasone propionate 250 µg twice-daily, *SD* standard deviation

Using a random-effects model, the mean difference between FF100 and FP250 in change from baseline in trough FEV_1_ was approximately −1.7 mL (95% confidence interval [CI] −80.4, +77.0); this difference was not statistically significant (*p* = 0.9664) (Fig. 1). FF100 was non-inferior to FP250 for the primary outcome measure, as the lower boundary of the 95% CI was greater than the pre-defined non-inferiority margin of −200 mL. The *Q* test and *I* ^2^ results indicated no statistically significant heterogeneity between the results from the two trials. A supporting meta-analysis using LS mean change from baseline in trough FEV_1_ also demonstrated no statistically significant difference between FF100 and FP250 (−7.9 mL [95% CI –87.1, +71.3], *p* = 0.8450), and non-inferiority of FF100 to FP250. Similar findings were obtained using a fixed-effects meta-analytic model (data not shown).

Meta-analytic approaches allow for the combined analysis of statistical data to obtain a more robust measure of treatment effect. Only two company-sponsored studies from the GSK FF clinical trial programme met the criteria for inclusion in this meta-analysis, having evaluated both FF100 and FP250 treatment arms. Combining the findings from the individual trials, the results of this meta-analysis demonstrated no statistically significant difference between FF100 and FP250, and non-inferiority of FF100 to FP250, in mean change from baseline in trough FEV_1_. With only two studies included in the meta-analysis, meta-regression to adjust for differences between patient populations was precluded; however, such adjustment was unlikely to have affected the results. Overall, the disparities between the two studies were minor and considered unlikely to have significantly impacted the trial outcomes. Furthermore, the *Q* test and *I* ^2^ results indicated no significant statistical heterogeneity between the trial results.

However, limitations of the analysis should be considered when interpreting these results. Firstly, both included studies, FFA109685 and FFA112059, were placebo-controlled trials that included FP250 only as a reference arm. Neither study was originally designed for investigating the equivalence or non-inferiority of FF to FP. Secondly, FF and FP were delivered via different inhalers in the two studies (ELLIPTA and Diskus/Accuhaler, respectively [5, 6]). Thirdly, the timing of the primary endpoint assessment differed between the two studies (8 weeks in FFA109685; 24 weeks in FFA112059); however, the rationale for comparing data from the two time points using a LOCF approach is supported by the following: (i) from a clinical perspective, steady state (FEV_1_ effect size) is already achieved by 8 weeks of treatment and (ii) trough FEV_1_ did plateau after 8 weeks in study FFA112059 [6]. Lastly, caveats associated with the limited sample size in this study should be noted. Meta-analyses including few studies have lower statistical power to detect the effect of an intervention, and preclude from performing publication bias tests, meta-regressions (to test for the presence of effect modifiers), subgroup analyses, and sensitivity analyses, for example. The analysis was limited by the number of patients with available data for inclusion (*N* = 433 across the two studies) and it is acknowledged that the degree of random error contributing to imprecise estimates of treatment effect may be reduced with a larger sample size.

## Conclusions

In this analysis, FF100 and FP250 demonstrated a comparable treatment effect on trough FEV_1_ in patients aged ≥12 years with persistent asthma; however, results interpretation should consider study design and methodological limitations.
